# In Vitro Study of the Anti-inflammatory and Antifibrotic Activity of Tannic Acid-Coated Curcumin-Loaded Nanoparticles in Human Tenocytes

**DOI:** 10.1021/acsami.3c05322

**Published:** 2023-05-02

**Authors:** Giuseppina Molinaro, Flavia Fontana, Rubén Pareja Tello, Shiqi Wang, Sandra López Cérda, Giulia Torrieri, Alexandra Correia, Eero Waris, Jouni T. Hirvonen, Goncalo Barreto, Hélder A. Santos

**Affiliations:** † Drug Research Program, Division of Pharmaceutical Chemistry and Technology, Faculty of Pharmacy, University of Helsinki, P.O. Box 56, Fabianinkatu 33, 00014 Helsinki, Finland; ‡ Department of Hand Surgery, University of Helsinki and Helsinki University Hospital, 00029 HUS Helsinki, Finland; § Translational Immunology Research Program, Faculty of Medicine, University of Helsinki, PL 4 (Yliopistonkatu 3), 00014 Helsinki, Finland; ∥ Medical Ultrasonics Laboratory (MEDUSA), Department of Neuroscience and Biomedical Engineering, Aalto University, 02150 Espoo, Finland; ⊥ Orton Orthopedic Hospital, Tenholantie 10, 00280 Helsinki, Finland; # Department of Biomedical Engineering, University Medical Center Groningen, University of Groningen, Ant. Deusinglaan 1, 9713 AV Groningen, The Netherlands; ¶ W. J. Kolff Institute for Biomedical Engineering and Materials Science, University Medical Center Groningen, University of Groningen, Ant. Deusinglaan 1, 9713 AV Groningen, The Netherlands

**Keywords:** nanoparticles, microfluidics, curcumin, tannic acid, tenocytes, inflammation

## Abstract

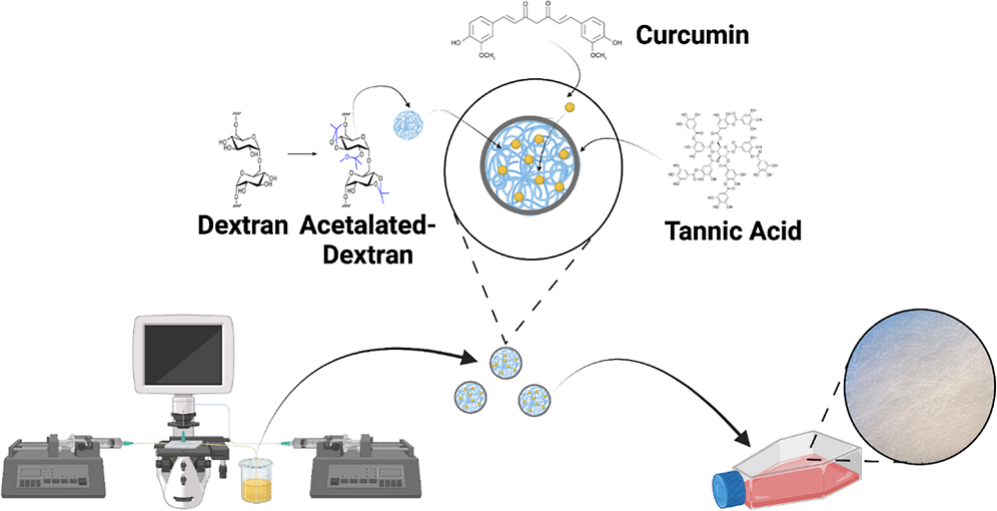

Tendinitis is a tendon disorder related to inflammation and pain, due to an injury or overuse of the tissue, which is hypocellular and hypovascular, leading to limited repair which occurs in a disorganized deposition of extracellular matrix that leads to scar formation and fibrosis, ultimately resulting in impaired tendon integrity. Current conventional treatments are limited and often ineffective, highlighting the need for new therapeutic strategies. In this work, acetalated-dextran nanoparticles (AcDEX NPs) loaded with curcumin and coated with tannic acid (TA) are developed to exploit the anti-inflammatory and anti-fibrotic properties of the two compounds. For this purpose, a microfluidic technique was used in order to obtain particles with a precise size distribution, aiming to decrease the batch-to-batch variability for possible future clinical translation. Coating with TA increased not only the stability of the nanosystem in different media but also enhanced the interaction and the cell-uptake in primary human tenocytes and KG-1 macrophages. The nanosystem exhibited good biocompatibility toward these cell types and a good release profile in an inflammatory environment. The efficacy was demonstrated by real-time quantitative polymerase chain reaction, in which the curcumin loaded in the particles showed good anti-inflammatory properties by decreasing the expression of NF-κb and TA-coated NPs showing anti-fibrotic effect, decreasing the gene expression of TGF-β. Overall, due to the loading of curcumin and TA in the AcDEX NPs, and their synergistic activity, this nanosystem has promising properties for future application in tendinitis.

## Introduction

Tendinitis is a tendon disorder associated with acute inflammation and pain, related to injuries or repetitive motion of the tendon over time.^1^ The tissue is hypocellular and hypovascular,^2^ so the healing process occurs with disorganized deposition of excessive extracellular matrix (ECM), resulting in scar formation that leads to decreased mechanical capacity of the tendon^3^ and risk of recurrence.^4,5^ Tenocytes are fibroblast-like cells whose main role is to control the turnover of ECM components and to respond to mechanical stimuli by modulating matrix composition and organization, in physiological condition, but also in acute and chronic disorder.^6^ Moreover, numerous cellular signaling pathways are involved in the adaptative response of tendon homeostasis, such as nuclear factor-kappa b (NF-κb), which is a critical pathway in the regulation of pro-inflammatory cytokines’ production and apoptosis. NF-κb is upregulated after tendon injury,^7,8^ as well as transforming growth factor-β (TGF-β),^9^ one of the key mediators in the development of fibrosis in tendon tissue.^10^

Conventional therapies, such as non-steroidal anti-inflammatory drugs, physiotherapy, and corticosteroid injections, currently aim to treat the symptoms and decrease local inflammation, but they do not solve the problem of fibrosis and histological change of the tendon structure.^1^ The systemic administration of anti-inflammatory drugs throughout the body via the bloodstream can lead to burst release, which cascades in several problems such as higher toxicity, reduced efficacy, and uneven distribution of the medication also to healthy tissue.^11,12^ In contrast, local delivery can provide more controlled and sustained release of drugs, reducing the risk of these issues.^13^ The drug can be delivered directly to the affected area, resulting in more targeted and efficient treatment.^12^ Additionally, a lower dose is typically needed, reducing the risk of toxicity and drug interactions.^12^

Polymeric-based nanoparticles (NPs) have been applied in the nanotechnology field as a drug delivery system with better bioavailability and controlled drug administration in the target site compared with traditional drugs.^14^ The advantages of these NPs are both related to the drug release profile and also to the possibility to deliver poorly water-soluble drugs using a lower dose with decreased side effects.^15^ Furthermore, NPs also allow the delivery of multiple compounds, favoring combined therapies.^16^ For these reasons, polymeric-based NPs can be used to treat inflammation and fibrosis in tendinitis since injectable NPs can extend the action time of the loaded drugs, improve the efficacy of the drug, and also decrease the toxicity, compared with conventional methods.^3^

More specifically, the pH-responsive acetalated-dextran (AcDEX) is a biocompatible polymer synthesized by the acetylation of dextran hydroxyl groups.^17^ The polymer is insoluble in water, and its degradation is triggered by an acidic pH.^18,19^ The acid-induced hydrolysis leads to the removal of acetal groups from AcDEX backbone, and the polymer is converted back to dextran, which is soluble in water.^20^ Since their first report by Chen et al.,^21^ AcDEX particles have been developed for the release of drugs under acidic conditions, as in inflammation sites. Due to the pH-responsive properties of AcDEX, injectable particles can enhance biological responses and improve the delivery in the target tissue.^22^ As a result of its hydrophobic properties, AcDEX is an advantageous polymer for drug encapsulation by precipitation, and its degradation at acidic pH facilitates drug release under acidified conditions such as during an inflammatory process.^18,23^

In this work, we develop a dual-acting nanoformulation, aiming to tackle inflammation and fibrosis during the healing process.

AcDEX NPs are loaded with curcumin and coated with tannic acid (TA). Curcumin, a natural polyphenol turmeric derived from *Curcuma longa*, was selected due to its anti-inflammatory capabilities. Curcumin plays a significant role in decreasing inflammation as its mechanism of action promotes the deactivation of NF-κb,^24^ indicating a key role in the inflammatory process and a decrease in the production of inflammatory mediators.^25^ TA is a compound that has shown both anti-inflammatory^26^ and anti-fibrotic properties, reducing the expression of TGF-β.^27^ Thus, we hypothesize that the co-delivery of curcumin and TA by our nanoformulation would tackle the problem of inflammation and excessive fibrosis in tendinitis. For this purpose, we fabricated the NPs using a microfluidic technique,^28^ in order to obtain particles with precise size and size distribution, leading to reduced batch-to-batch variations,^29^ and to facilitate a future clinical translation.

**Figure 1:**
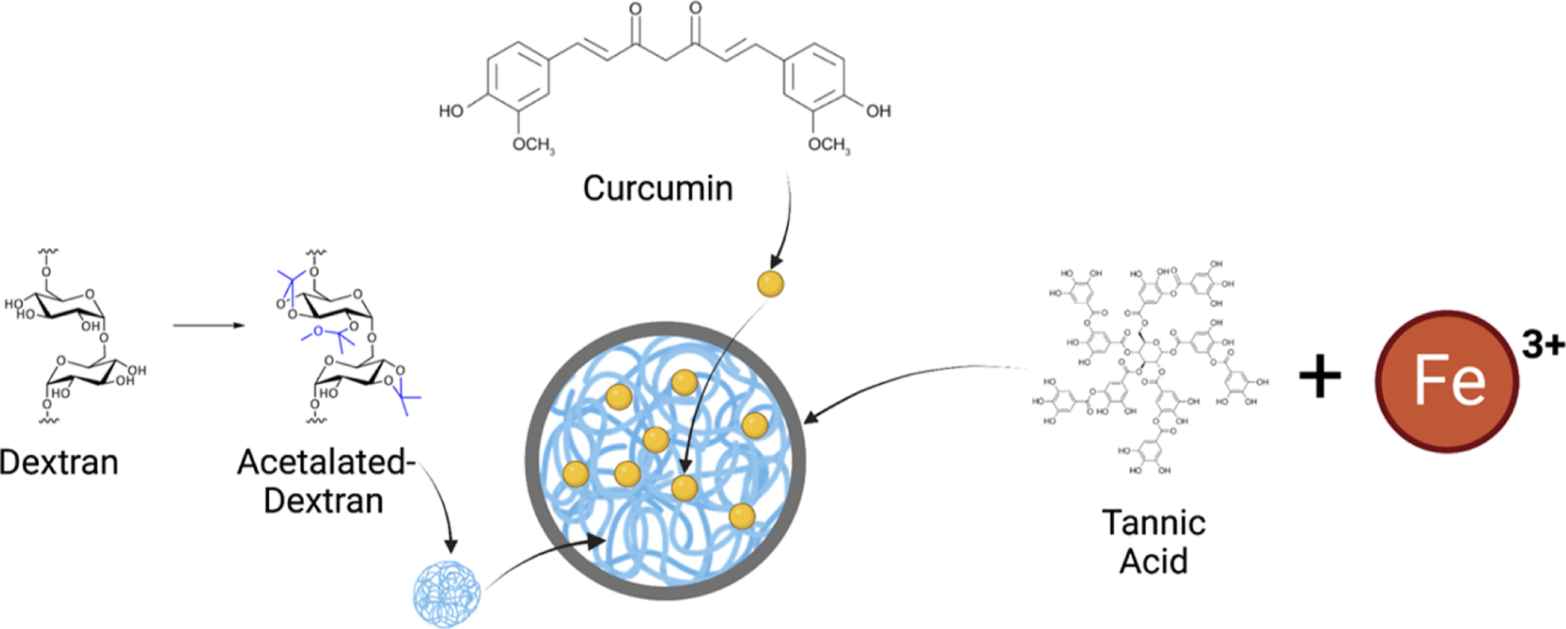
Schematic illustration of AcDEX NPs loaded with curcumin and coated with the tannic acid-Fe^3+^ complex (TA-coated NPs). Image partially created with Biorender.com.

The main aim of this work was to fabricate AcDEX NPs loaded with curcumin (drug-loaded NPs) and coated with TA (TA-coated NPs, shown in Figure 1) and to evaluate the in vitro cytocompatibility of the developed NPs and the cell–NPs interactions in human tenocytes and macrophages, cell lines involved in the inflammatory process of the tendon. Finally, we investigated the role played by the developed nanosystem in the inhibition of inflammatory and fibrotic processes in human tenocytes.

## Results and Discussion

### Physicochemical Characterization of NPs

First, we modified dextran in AcDEX (Figure S1), followed by the optimization in microfluidics to obtain AcDEX and drug-loaded NPs using the nanoprecipitation process in a glass-capillary device already developed by Liu et al.^30^ For the optimization of the parameters in the microfluidic device, first, we used AcDEX without curcumin to develop the empty AcDEX NPs. A solution of ethanol and 1% Poloxamer-188 in Milli-Q water (pH 8) has been selected as the inner and outer fluid, respectively. After different trials (Table S1), at the flow rate of 2 mL/h for the inner fluid and of 60 mL/h for the outer fluid, we achieved AcDEX NPs with a uniform size of 191.2 ± 21.9 nm and a polydispersity index (PDI) below 0.2; therefore, we translated these parameters for the production of the drug-loaded NPs.

The drug-loaded NPs were prepared by dissolving curcumin, together with AcDEX, in ethanol, a solvent suitable for the dissolution of both compounds,^18,31^ to form the inner phase which was then precipitated in 1% poloxamer-188 in water. Then, to obtain the TA-coated NPs, a solution of TA and Fe^3+^ was added to 0.5 mg of drug-loaded NPs, as described by Torrieri et al.^32^ The color of the NPs in suspension turned from yellow to gray, demonstrating that the coating took place on the surface of the particles after a few seconds. The presence of the coating in the formulation is important for the loading of the TA, due to its anti-fibrotic effect, and also for improving the stability of the particles in cell culture media.

**Figure 2:**
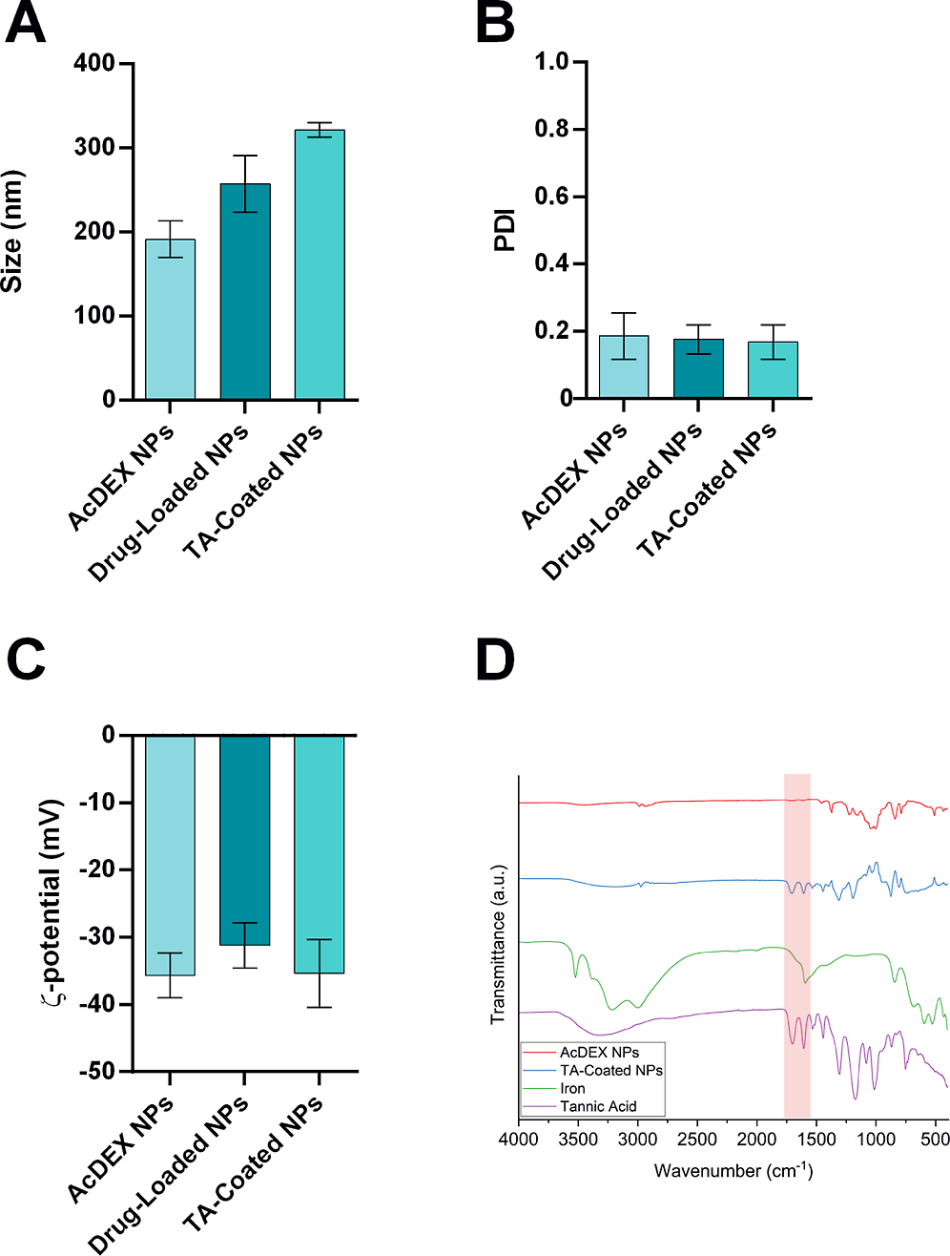
Physicochemical characterization of the optimized NPs using DLS and ELS in terms of (A) size (nm), (B) PDI, and (C) ζ-potential (mV). Drug-loaded NPs and TA-coated NPs are AcDEX NPs loaded with curcumin and AcDEX NPs loaded with curcumin and coated with TA, respectively. Results are presented as mean ± s.d. (*n* ≥ 3 independent batches of particles prepared in different days). (D) ATR–FTIR spectra for the chemical composition of the NPs surface.

The size and homogeneity of the particles (Figure 2A,B) were studied by dynamic light scattering (DLS) and the ζ-potential by electrophoretic light scattering (ELS) (Figure 2C). As shown in Figure 2A, the size of the TA-coated NPs (321.1 ± 8.7 nm) increased compared to the AcDEX and the drug-loaded NPs (257.1 ± 33.7 nm), suggesting that a layer of coating had deposited on the surface. In addition, drug-loaded NPs also exhibit an increase in size compared to AcDEX NPs due to the presence of curcumin within the polymer. The PDI of the NPs did not show any statistically significant differences between the particles and the average value always remained below 0.2, indicating the low polydispersity of the different samples analyzed (Figure 2B). The surface charge of the NPs remained negative (Figure 2C) due to the presence of the AcDEX polymer,^33^ and the addition of TA-Fe^3+^ did not alter the overall surface charge of the NPs.

We evaluated the successful coating of the NPs with the TA-iron complex by attenuated total reflectance–Fourier transform infrared (ATR–FTIR) of the TA and iron alone and the NPs before and after the coating. As shown in Figure 2D, after the coating process on the NPs’ surface, there are 2 bands corresponding to the stretching of the aromatic system of the TA around 1600 cm^–1^ (CC) and the stretching of the carbonyl of the ester groups of the TA around 1700 cm^–1^ (CO).^36^

**Figure 3:**
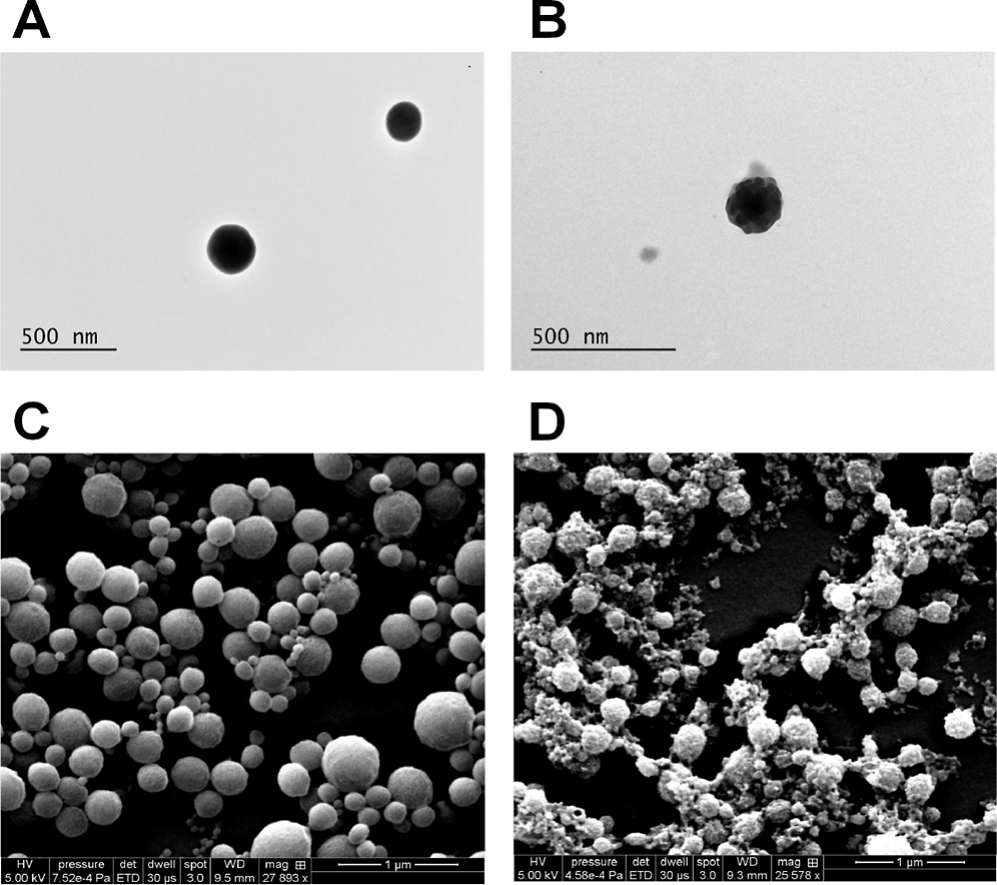
TEM and SEM images displaying the changes in the morphological structure of the NPs before (A–C) and after (B–D) the TA-coating, respectively. Scale bars are reported in each image.

To further confirm the success of the coating process, the morphology of the particles was studied by transmission electron microscopy (TEM) and scanning electron microscopy (SEM). In Figures 3 and S2, the difference between drug-loaded and coated NPs is clearly seen: the surface of the TA-coated NPs is irregular and blunt (Figure 3B–D) when compared to the regular surface of the NPs (Figure 3A–C). The concentration of TA and Fe^3+^ was studied by indirect quantification using Folin–Ciocalteu’s method^34^ and isothiocyanate colorimetry,^35^ respectively, and 248.6 μg of TA and 22.5 μg of Fe^3+^ were quantified on the surface of 1 mg of NPs.

### Stability and Drug Release Studies

**Figure 4:**
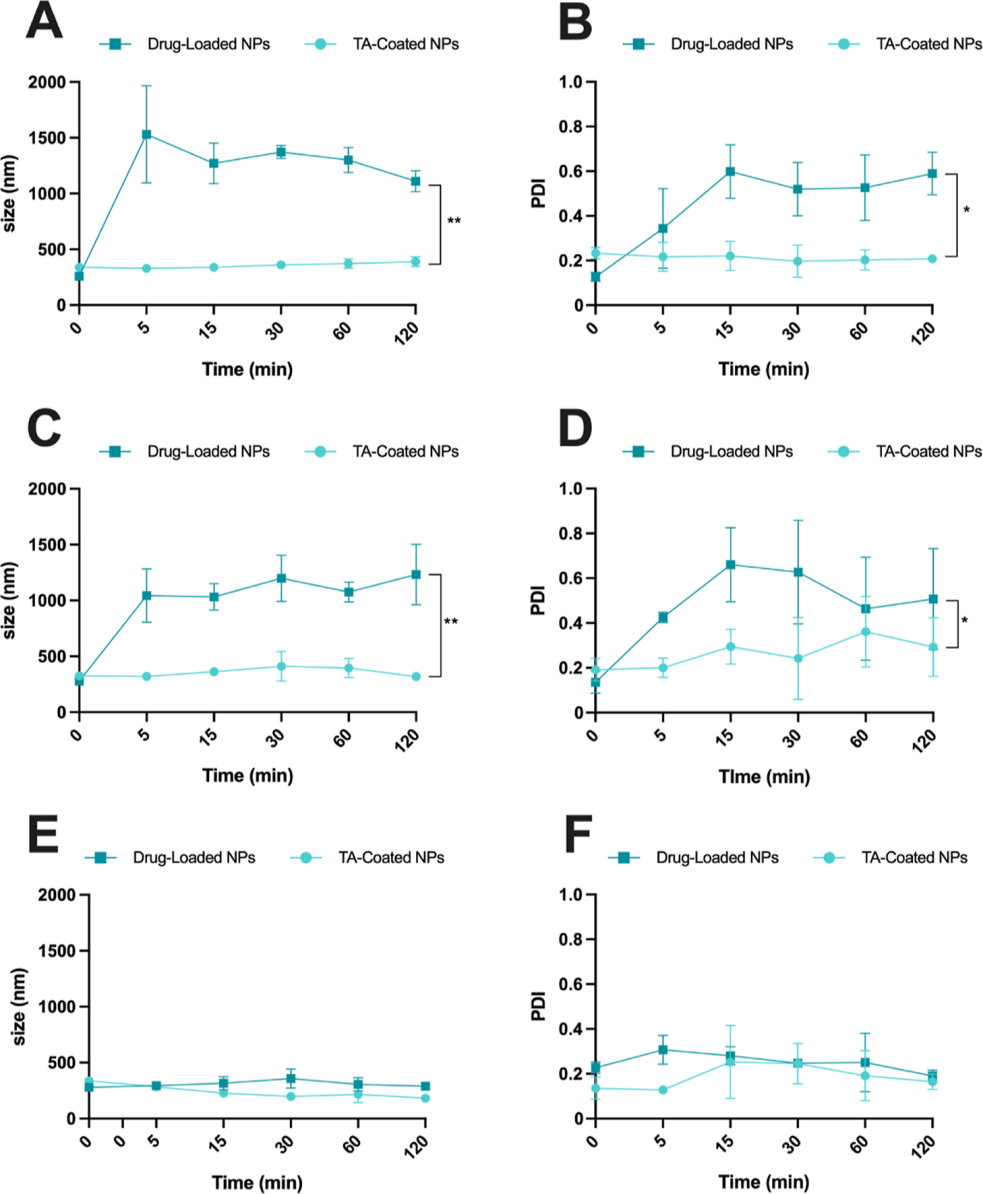
Colloidal stability of NPs in tenocytes’ cell culture medium in terms of (A) size and (B) PDI of the tested NP-formulations. Colloidal stability of NPs in KG-1 cell culture medium in terms of (C) size and (D) PDI of the tested NP-formulations. Colloidal stability of NPs in 5.4% of sucrose in terms of (E) size and (F) PDI of the tested NP-formulations. Results are presented as mean ± s.d. (*n* ≥ 3 independent batches of particles prepared in different days). A paired Student’s *t*-test was used for statistical analysis, and *p* values were set at probabilities *p < 0.05 and **p < 0.01, comparing the profile size vs time or PDI vs time for both samples.

The colloidal stability of the developed NPs was investigated in tenocytes and KG-1 macrophages’ cell culture media, Dulbecco’s modified Eagle’s medium (DMEM-F12) and Iscoved’s modified Dulbecco’s medium (IMDM), respectively, and in an isotonic solution of sucrose (5.4%, w/v). We decided to test the stability in those different media since the experiments in vitro were conducted in the medium of these two cell lines, and the isotonic solution of sucrose is often used for NPs-formulation testing in vivo. When particles were tested in DMEM-F12 and IMDM, containing 10% of fetal bovine serum (FBS), the drug-loaded NPs showed an increase in size (Figure 4A–C) and PDI (Figure 4B–D) over time, while the TA-coated NPs were stable, with almost no increase in size and good polydispersity up to 2 h. These results showed that the TA-Fe^3+^ complex gave stability to the NPs by reducing interaction with serum proteins in the medium,^32^ compared with the drug-loaded NPs, in which the surface is covered only by the AcDEX. Moreover, the data of the drug-loaded NPs (Figure 4A–C) displayed a large standard deviation between the replicates, and the PDI data (Figure 4B–D) demonstrated that the particles are aggregating, thereby also influencing the size values reported from the instrument.

The stability was also monitored in an isotonic sucrose solution, and both nanosystems showed good colloidal stability since there was no significant increase in terms of size and PDI (Figure 4E,F).

Next, the in vitro release profile of curcumin from the NPs was evaluated at pH 5.5 and 7.4, in a synovial-mimicking fluid,^37^ to mimic the condition of inflammatory and healthy tissue microenvironment.

**Figure 5:**
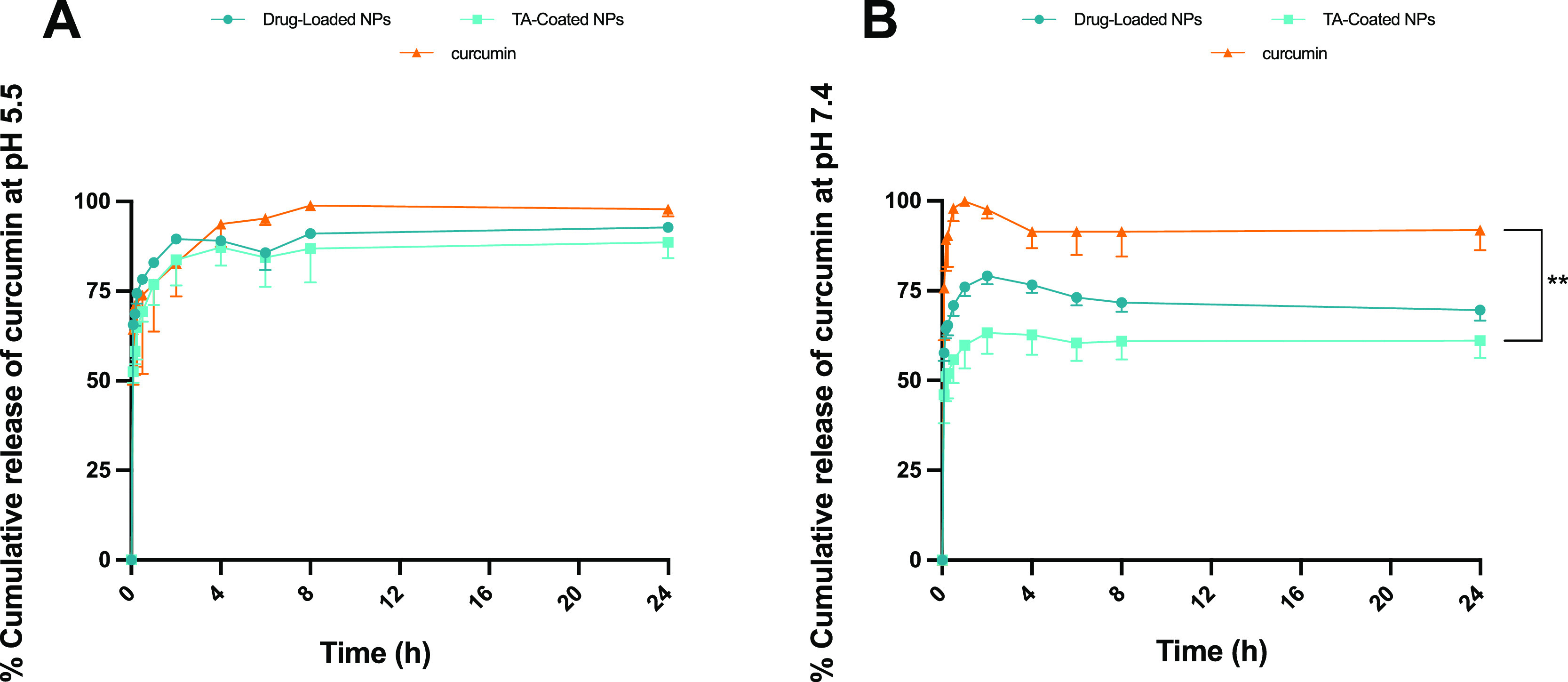
Evaluation of drug-release profile in sink conditions of curcumin from drug-loaded and TA-coated NPs, in a synovial-mimicking fluid at (A) pH 5.5 and (B) pH 7.4, kept at 37 °C and under stirring at 300 rpm. Results are presented as mean ± s.d. (*n* ≥ 3), and the samples were analyzed with ordinary one-way ANOVA, followed by a Dunnett post-hoc test, setting the probabilities at **p < 0.01, comparing the release vs time profile between the NPs and curcumin. The cumulative release of curcumin, drug-loaded, and TA-coated NPs up to 2 h is shown in Supporting Information (Figure S3).

Before determining the release profile, a known quantity of NPs was weighed, and the loading degree (LD %) and encapsulation efficiency (EE %) of both nanosystems were determined using high-performance liquid chromatography (HPLC) (Table S2). As shown in Figure 5A, for both drug-loaded and TA-coated NPs, there is an initial fast drug release of up 2 h, where almost 80% of the payload of curcumin is released from the loaded NPs and around 75% from the coated ones. Thereafter, after 6 h, the curcumin release is constant, and the curve reaches a plateau up to 24 h. The cumulative release of curcumin, drug-loaded, and TA-coated NPs up to 2 h is shown in Figure S3 of the Supporting Information.

This similar profile in both NPs can be explained by the fact that the degradation of the AcDEX polymer is pH-dependent,^17^ so at pH 5.5, hydrolysis of the acetal groups on the surface of the NPs takes place. Likewise, the TA-Fe^3+^ complex is disassembled at acidic pH,^32,38^ as the hydroxyl groups of the TA are protonated, and this leads to a disintegration of the coating. In Figure 5B, in the synovial-mimicking fluid at pH 7.4, an initial burst release is observed for drug-loaded and TA-coated NPs, which is decreased in rate if compared to that at pH 5.5. This can be outlined by the fact that some curcumin can be adsorbed on the surface of NPs, and it is the first to be released, while later, the release depends on the degradation of AcDEX in the respective mediums at different pH, and these nanosystems have a preferential release in the inflammatory environment, compared with the one under the physiological condition. Furthermore, the release medium contained 1% of Tween in order to ensure the stability of curcumin in the sink condition, and this increases the release rate of the drug.

### Cytocompatibility Studies

The cytocompatibility of the NPs was evaluated on human tenocytes and KG-1, which were used as a model of macrophages recruited during the inflammatory process, where they play a key role.^6^ The human tenocytes were isolated from a 58 year-old patient due to the rupture of the extensor *pollicis longus* tendon: the sample is derived from the extensor *indicis proprius*.

The cell viability was quantified after incubation for 24 h with the NPs, considering the time point presenting the maximum release of curcumin. Curcumin alone was also tested, as well as TA, Fe^3+^, and the TA-Fe^3+^ complex (Figure S4). The cells in medium and Triton-X were used as the negative and positive controls, respectively. The concentrations of NPs tested were in a range between 50 and 500 μg/mL in order to assess how the different NP-concentrations affected the cell viability.

The concentrations of curcumin tested were calculated for each concentration of the NPs taking into account the LD of the TA-coated NPs, in order to compare the cytocompatibility of the same amount of compound, delivered in the NPs and alone. The same strategy was used for the concentrations of TA, Fe^3+^, and the TA-Fe^3+^ complex tested in both cell lines. The cytotoxicity was tested evaluating the effect of the NPs and the compounds on the cellular viability by an adenosine triphosphate (ATP)-luciferase assay (CellTiter-Glo luminescence assay).^37^

**Figure 6:**
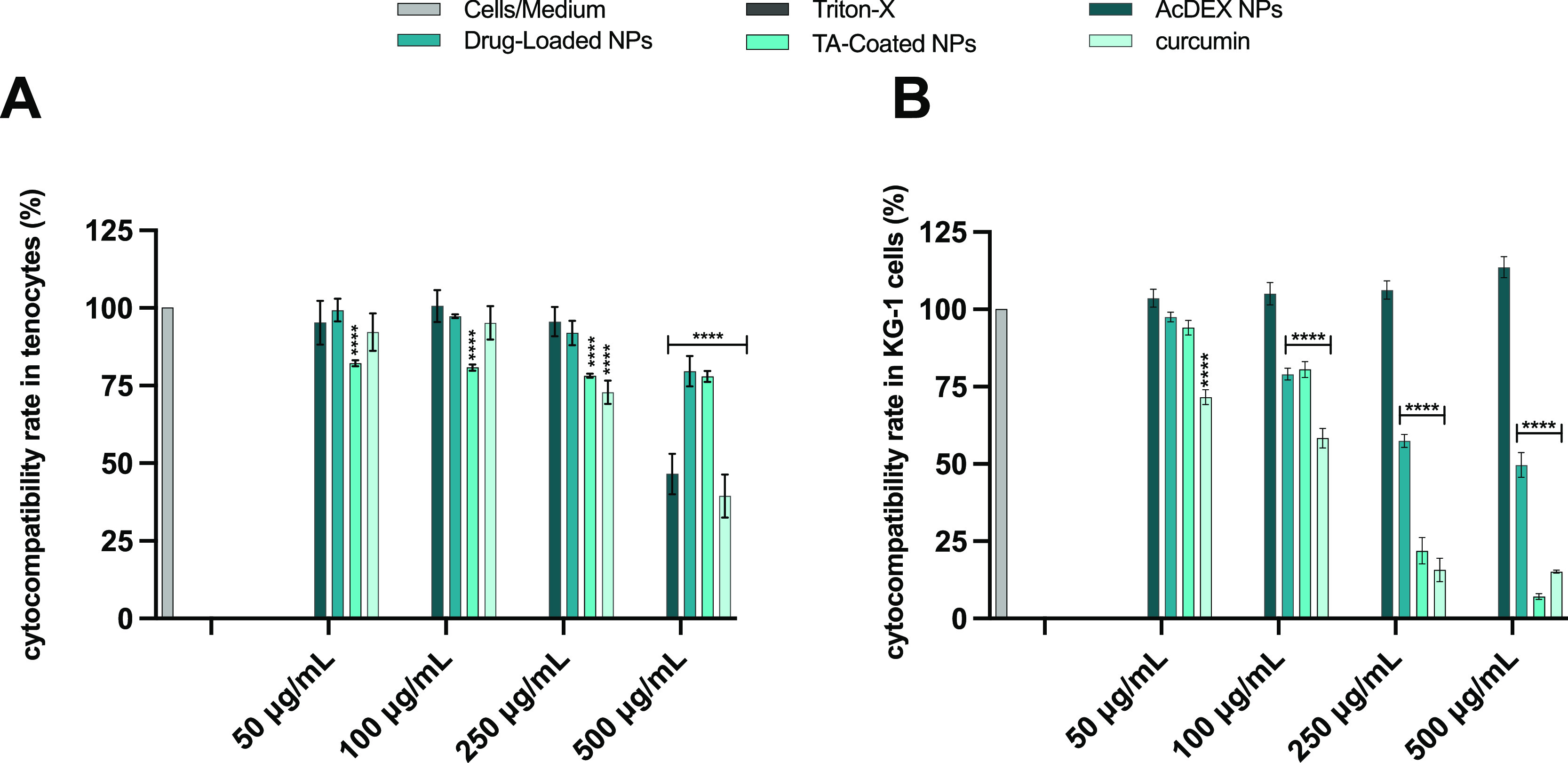
Cell viability studies of AcDEX, drug-loaded, TA-coated NPs, and curcumin on (A) human primary tenocytes and (B) KG-1 macrophages after 24 h of incubation. The concentrations of curcumin tested were calculated for each concentration of NPs taking into account the LD of the TA-coated NPs. Cell culture media and Triton-X 100 (1%) represented negative and positive controls, respectively. Results are presented as mean ± SD (*n* ≥ 3), and the samples were analyzed with ordinary two-way ANOVA, followed by a Dunnett post-hoc test, setting the probabilities at ***p < 0.001 and ****p < 0.0001, comparing all the samples to the negative control.

As shown in Figure 6A, the AcDEX NPs reduced the cytocompatibility at the highest dose (500 μg/mL) in primary human tenocytes. In the case of KG-1 (Figure 6B), the AcDEX NPs are not toxic up to the highest concentration. Drug-loaded NPs showed no significant decrease in cytocompatibility in human tenocytes, but in KG-1, it displayed a dose-dependent toxicity related to the mode of action of curcumin, which interferes with the replication process of these tumor cells, decreasing the expression of Nf-κb.^39^ Curcumin solution showed the same profile on KG-1 as the drug-loaded NPs (Figure 6B), demonstrating that in the drug-loaded NPs, the drug is responsible for decreasing the cell viability. The TA-coated NPs showed no increase in concentration-related toxicity on human tenocytes, maintaining the cell viability at around 80%, whereas in KG-1, the toxicity is dose-dependent (Figure 6A,B). This can be explained as TA promotes the downregulation of P3K-AKT,^40^ an important pathway for the proliferation of these tumor cells involved in acute myelogenous leukemia.^41^ Therefore, considering the cell viability of TA-coated NPs, we decided to test in vitro the concentration of 100 μg/mL, excluding higher concentrations that may cause cell toxicity.

### Quantitative Uptake in Human Tenocytes

**Figure 7:**
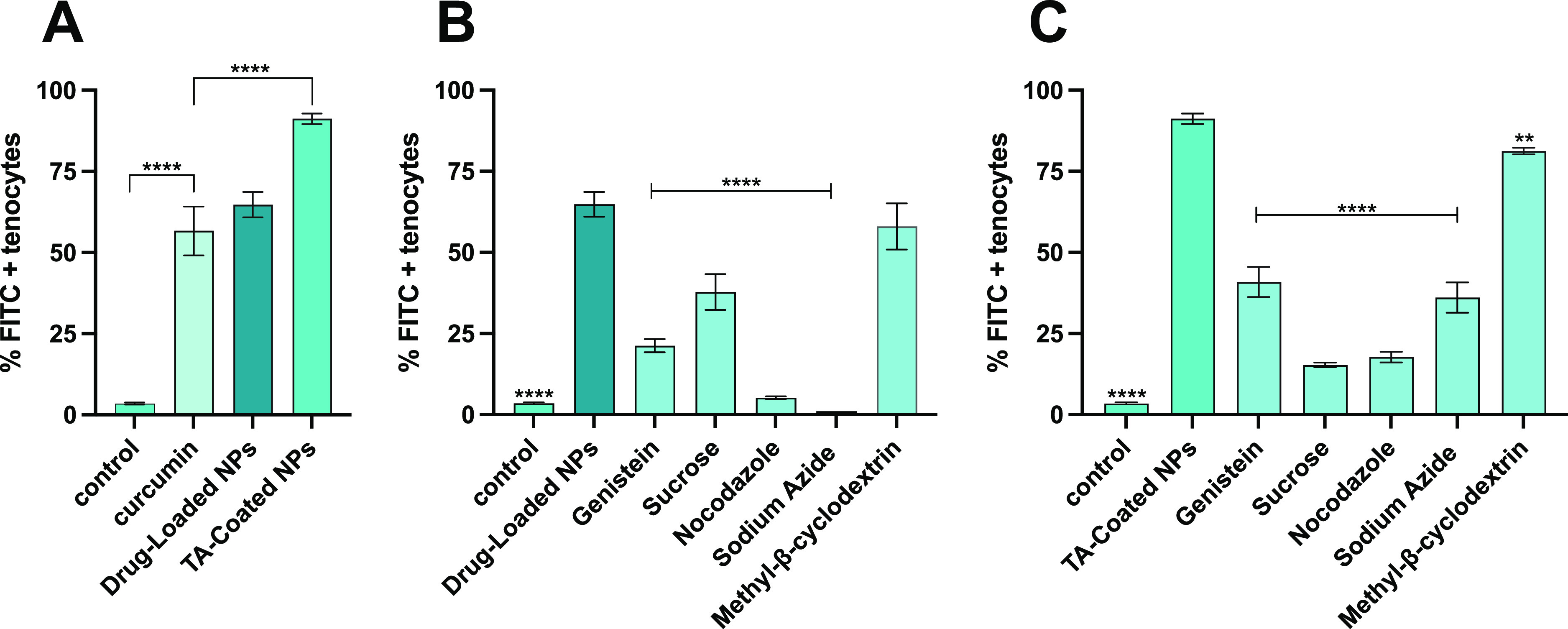
Quantitative cell uptake and cell uptake mechanism studies on human primary tenocytes using a flow cytometer. (A) Cells were incubated for 3 h with curcumin, drug-loaded, and TA-coated NPs. Cells were pre-treated with different inhibitors and then incubated for 3 h with drug-loaded (B) or TA-coated (C) NPs to study the mechanism of endocytosis by a flow cytometer. Results are presented as mean ± SD (*n* ≥ 3), and the samples were analyzed with ordinary one-way ANOVA, followed by a Dunnett post-hoc test, setting the probabilities **p < 0.01 and ****p < 0.0001, comparing in (A) all the samples vs curcumin, (B) all the samples vs the drug-loaded NPs, and in (C) all the samples vs the TA-coated NPs.

The interaction between the NPs and cells was evaluated quantitatively in human primary tenocytes and KG-1 (Figure S5). For the quantitative uptake, cells were treated with curcumin, drug-loaded, and TA-coated NPs for 3 h, and then the quantitative cell-uptake was evaluated by flow cytometry. Curcumin emits fluorescence in the fluorescein isothiocyanate (FITC) channel when excited by a 488 nm laser, thereby curcumin’s fluorescence signal was used to measure the uptake by flow cytometry. As shown in Figure 7A, the percentage of FITC + tenocytes for the curcumin and drug-loaded NPs is around 55%; in contrast, it is 90% for the TA-coated NPs, showing a marked increased uptake into cells.

In order to study the quantitative uptake in KG-1 steady state (M0) and activated macrophages (M1), cells were treated with 100 ng/mL of lipopolysaccharide (LPS) and incubated for 24 h. To confirm the switching of phenotype from M0 to M1, the expression of the marker CD86 was measured by a flow cytometer, as shown in Figure S6. Then the cells–NPs interaction was performed as described above. As shown in Figure S5, both drug-loaded and TA-coated NPs are preferentially internalized by M1 macrophages, with an increase around 50%, when compared to M0 macrophages. These results are in agreement with the fact that M1-activated macrophages have higher phagocytosis capacity when compared with the M0-phenotype.^42^

The mechanism used by the cells to internalize the particles was also studied by pre-treating the human tenocytes with different inhibitors of endocytosis, before adding the NPs, since this is the main mechanism that NPs use to enter cells.^43^ The cell cytotoxicity of different concentrations of these inhibitors (Table S4) was performed (Figure S7) and, according to the results, the inhibition conditions used were not toxic to the cells.

Genistein is an isoflavone that inhibits tyrosine kinase by disrupting the actin network at the site of endocytosis and preventing the caveolae-dependent mechanism.^44^ Clathrin-mediated uptake was inhibited by sucrose, an agent that prevents the recycling of clathrin to the plasma membrane, thus inhibiting clathrin-mediated endocytosis.^45^ Sodium azide was used as an inhibitor of active transport, since it interferes with the production of ATP, inhibiting the cytochrome C oxidase.^46^ Nocodazole served as the suppressor of microtubules’ polymerization,^47^ and methyl-β-cyclodextrin was used for its mechanism of disruption of lipid rafts by decreasing the cholesterol component.^48^

As shown in Figure 7B, nocodazole and sodium azide play a key role in the inhibition of drug-loaded NPs uptake, showing a decrease of more than 90%; on the other hand, genistein and sucrose also contribute to the decrease of NPs internalization, confirming that more endocytosis processes are involved in NPs–cells interactions. Figure 7C shows how sucrose and nocodazole predominantly inhibit the internalization of TA-coated NPs, suggesting that the main uptake mechanisms are related to clathrin-mediated endocytosis and actin polymerization. Also, energy-dependent pathways and caveolin-mediated endocytosis showed a decrease in the TA-coated NPs uptake by around 50%, demonstrating that these mechanisms are also involved in particles’ internalization.

### Qualitative Uptake in Human Tenocytes

**Figure 8:**
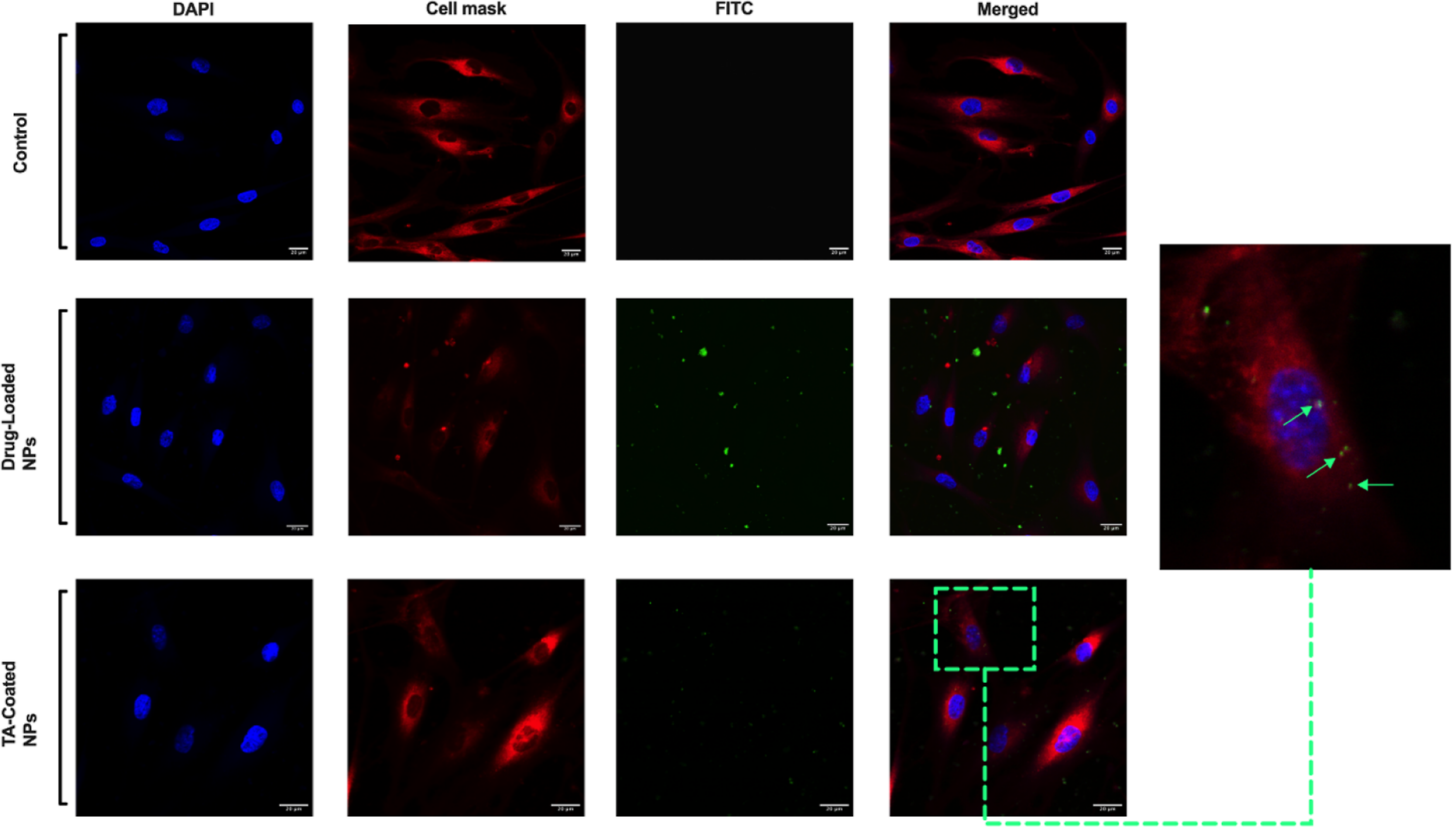
Qualitative uptake studies of NPs in human tenocytes. The cell uptake was evaluated by confocal fluorescence microscopy after incubation with the NPs for 1 h at 37 °C. The NPs, loaded with curcumin, were visualized in FITC (green channel), while cells were stained with DAPI (nuclei, blue channel) and CellMask (cell membrane, red channel). Scale bars are shown in each image.

The qualitative uptake was also performed on human tenocytes by imaging the samples with confocal microscopy. To perform this study, cells were incubated with the drug-loaded and TA-coated NPs for 1 h at 37 °C in order to evaluate the interaction between NPs-cells. Cells alone were used as a negative control. After incubation, the cell membrane was stained with CellMask Deep Red, then cells were fixed with a solution of paraformaldehyde (PFA), and the nuclei were stained with 4′,6-diamidino-2-phenylindole (DAPI). As shown in Figure 8, the TA-coated NPs interact more with human tenocytes compared to the drug-loaded NPs, in which the majority of particles can be observed outside the cells. Supporting this observation is the fact that TA has been proven to have a particular affinity for collagen I,^49^ an essential component of the ECM produced by tenocytes.^49^ Therefore, TA-coated NPs may achieve higher internalization in these cells, and this may be in accordance with the flow cytometer quantitative uptake data, where the percentage of uptaken NPs was higher for TA-coated compared to that for drug-loaded ones. Moreover, as shown in Figure 8, the drug-loaded NPs might be aggregated outside the cells, and this would be in agreement with the data of the stability of these particles in human tenocytes’ media (Figure 4A).

### Anti-inflammatory Effect of Curcumin and Anti-Fibrotic Effect of TA

**Figure 9:**
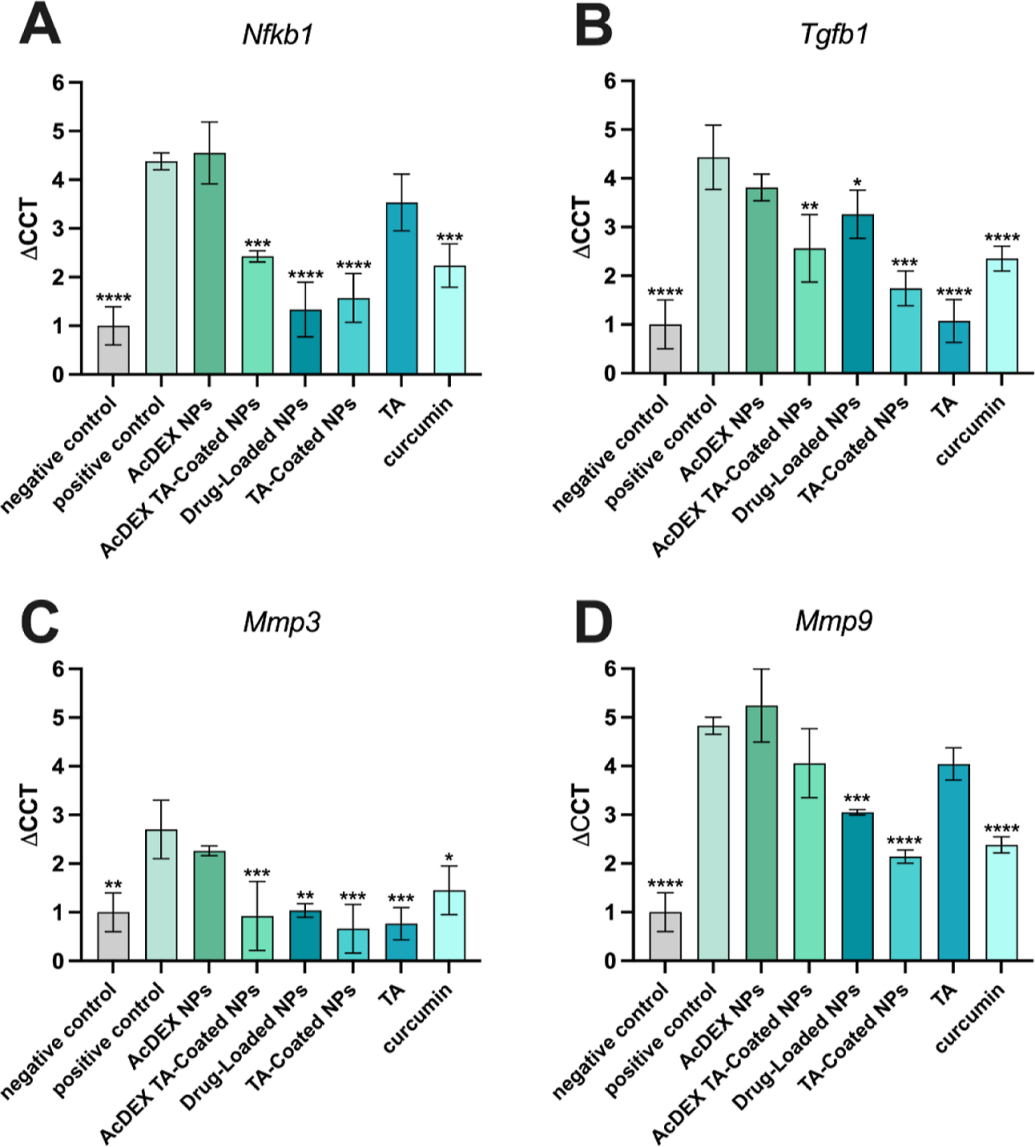
Evaluation of expression of pro-inflammatory and pro-fibrotic genes by RT-qPCR. The properties of curcumin and TA have been evaluated with AcDEX, AcDEX TA-coated, drug-loaded, and TA-coated NPs, as well as the curcumin and TA alone, by quantification of the gene expression of (A) *Nfkb1*, (B) *Tgfb1*, (C) *Mmp3*, and (D) *Mmp9*. Results are represented as fold increase values compared to the control ± s.d. (*n* ≥ 3). An ordinary one-way ANOVA followed by a Dunnett post-hoc test was used for the statistical analysis. The significance levels of the differences were set at the probabilities of *p < 0.05, **p < 0.01, ***p < 0.001, and ****p < 0.0001 for comparison with the positive control.

The anti-inflammatory effect of curcumin and the anti-fibrotic effect of TA were evaluated by real-time quantitative polymerase chain reaction (RT-qPCR). In this study, the synergistic effect of these two compounds in reducing the pro-inflammatory gene NF-κb and the pro-fibrotic gene TGF-β was investigated. In addition, the expression of metalloproteinases 3 (MMP-3) and 9 (MMP-9) was also studied, as they play an important role in the degradation of the tendon matrix, leading to structural changes and contributing to the pain and decreased functions, commonly associated with tendinopathy.^50^ Human tenocytes were pre-treated with 250 ng/mL of LPS for 24 h and then AcDEX, AcDEX TA-coated, drug-loaded, and TA-coated NPs, as well as curcumin, TA, and DMEM-F12 as control were added for 24 h. The *18S* gene was used as a housekeeping gene control. NF-κb is considered a critical pathway in the regulation of pro-inflammatory cytokines’ production and apoptosis,^7,24^ and TGF-β is one of the key mediators in the development of fibrosis,^10^ and they are both upregulated in tendinitis. As shown in Figure 9, cells treated with TA-coated NPs showed a decrease in the expression of *Nfkb1*, *Tgfb1*, *Mmp3*, and *9* when compared with positive control. In particular, the decrease in the gene expression of NF-κb and TGF-β was around 2-fold when the cells were treated with these NPs. On the other hand, also drug-loaded NPs showed a significant decrease in NF-κb gene expression since curcumin is the main compound involved in the process.^24^ As shown in Figure 9A,B, AcDEX TA-coated NPs also contribute to the decrease in the expression of *Nfkb1* and *Tgfb1* as they have TA on the surface. The 1.5-fold decrease of *Mmp3* and 2-fold decrease of *Mmp9* (Figure 9C,D) by the TA-coated NPs confirmed the role of curcumin in the decreasing metalloproteinase expression due to its mechanism of down-regulation of NF-κb.^51,52^ These results showed that TA and curcumin, delivered into NPs, can inhibit the expression of pro-inflammatory and pro-fibrotic genes in vitro and may have a potential effect in repairing inflamed tissues.

## Conclusions

In this work, we developed, using a microfluidic technique, AcDEX-based NPs loaded with curcumin and coated with TA. As a proof of concept, we investigated the therapeutic potential of TA-coated NPs, aiming at decreasing inflammation and fibrosis. The NPs were homogeneous in size, stable in cell culture media, and in the isotonic solution of 5.4% sucrose, with an increase in stability when coated with TA. The TA-coated NPs showed a burst release profile during the first 2 h in the synovial-mimicking fluid, where the release of curcumin is favored in the inflamed condition. The TA-coated NPs were compatible at low concentrations in KG-1 and in a range between 100 and 500 μg/mL on human tenocytes, showing good biosafety. The TA-coated NPs were also preferentially internalized by human tenocytes, showing enhanced cell-uptake when compared to curcumin or the drug-loaded NPs.

The efficacy of this nanosystem was demonstrated by RT-qPCR, in which the curcumin loaded in the particles showed anti-inflammatory properties, reducing NF-κb expression by more than 60%, while the TA coating on the NPs showed its anti-fibrotic activity decreasing by more than half the gene expression of TGF-β. Overall, due to the synergistic anti-inflammatory and anti-fibrotic activity, this nanosystem has promising properties for application in tendinitis treatment. Further research is needed to fully understand the potential benefits and limitations of this approach and to determine its efficacy in human patients with tendinopathy.

## Materials and Methods

### Synthesis of Drug-Loaded NPs and Coating with TA

Acetalated dextran (AcDEX) NPs were prepared by nanoprecipitation with a microfluidic device, as described elsewhere.^33^ Briefly, two glass capillaries were assembled on a glass slide with a 3D co-flow geometry; the inner capillary, with a diameter of 1.0 mm tapered to a tip of approximately 100 μm, was aligned into a bigger one of 1.10 mm (World Precision Instruments Inc. USA) and fixed with 5 min epoxy resin glue. AcDEX polymer (Figure S1), synthesized as described in Supporting Information, (10 mg) was dissolved in ethanol (900 μL), and subsequently, 100 μL of a solution (5 mg/mL in ethanol) containing curcumin (TCI, Japan) was added to the previous one. A solution of 1% Poloxamer-188 (BASF, Germany) was prepared in Milli-Q water (pH 8), and the two miscible liquids were pumped (PHD 2000, Harvard Apparatus, USA) into the chip from syringes connected with a polyethylene tube with a constant flow rate (flow rate inner phase: 2 mL/h; outer phase 60 mL/h). These drug-loaded NPs were pelleted by centrifugation 154,324*g*, 35 min (Optima L-100 XP Ultracentrifuge, Beckman Coulter), collected, and washed twice with Milli-Q water. The drug-loaded NPs were then coated with TA.^38^ 0.5 mg of NPs was resuspended in 500 μL of Milli-Q water, and 5 μL of a solution of TA (Sigma-Aldrich, USA), 40 mg/mL in Milli-Q water, was added and vortexed for 30 s; then, 5 μL of iron (Fe^3+^ chloride hexahydrate, Sigma-Aldrich, USA), 6 mg/mL in Milli-Q water, was added, and the solution was also vortexed for other 30 s. TA-coated NPs were centrifuged at 11,200*g* × 4 min, and the supernatant was collected for the detection of the two compounds by indirect quantification with Folin–Ciocalteu’s method^34^ and isothiocyanate colorimetry,^35^ respectively.

### Physicochemical Properties of NPs and Stability Studies

Size, PDI, and ζ-potential were characterized using DLS and ELS using a Zetasizer nano instrument (Malvern Instrument Ltd., UK). Briefly, 50 μL of NPs solution (1 mg/mL) was diluted in 950 μL of Milli-Q water before each measurement in a disposable polystyrene cuvette (SARSTEDT AG & Co., Germany) for the quantification of size and PDI. About 750 μL of a solution was then transferred in a disposable folded capillary cell (DTS1070, Malvern, UK) to quantify the ζ-potential.

Freeze-dried NPs were analyzed by ATR–FTIR spectroscopy (Bruker Vertex 70, Bruker, USA), and the spectra were recorded in the range of 4000 to 480 cm^–1^, with a resolution of 2 cm^–1^ using OMNIC software.

The shape and surface of these nanosystems were studied using TEM (Jeol JEM-1400, Japan) by adding 5 μL of a solution containing NPs (0.5 mg/mL) on a carbon-coated copper grid (Electron Microscopy Science, FCF 200-CU Mesh Copper, USA), left to dry at RT overnight before imaging.

The external morphology of the NPs was also characterized using SEM (SEM Quanta FEG 250, FEI, USA) at an accelerating voltage of 5.0 kV. For this study, samples were dried overnight, placed on a silica substrate, and coated with a 10 nm platinum layer.

Stability studies of NPs were evaluated by incubating particles in the cell medium used for human tenocytes, KG-1 macrophages, and in a solution of 5.4% (w/v) of sucrose (VWR Chemicals). 0.6 mg of NPs was resuspended in 200 μL of Milli-Q water and added in 1.3 mL of different stability media and stirred (150 rpm) at 37 °C. After different time points, 200 μL was taken from each sample, diluted in 800 μL of Milli-Q water, and measured by DLS.

### Quantification of Drug Loading (Curcumin and TA-Iron) and Drug Release

To determine the LD and EE of curcumin, a known amount of particles was dissolved in ethanol, and the LD was quantified using eq 1 and the EE using eq 2:
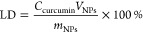
1where *C*_curcumin_ is the concentration of curcumin quantified (in μg mL^–1^), *V*_NPs_ is the volume of NPs (in mL), and *m*_NPs_ is the mass of NPs (in μg).
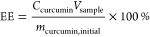
2where *V*_sample_ is the volume of the sample (in mL) and *m*_curcumin,initial_ is the initial amount of curcumin added (in μg).

The quantification was done by HPLC, (Agilent 1200 series, Agilent Technologies, USA). A Gemini 5 μm reversed-phase column (100 × 4.6 mm, Phenomenex) was used with a mobile phase (45:55) of 0.2% phosphoric acid and acetonitrile (ACN). The flow rate used was 1 mL/min, the injection was 10 μL, and the wavelength was set at λ = 280 nm.

TA and iron were quantified by an indirect method. After coating the NPs and centrifuging them, the supernatant was acidified with 50 μL of HCl 1 M to destroy the coordination complex. Then, the two compounds were determined as described below.^32^ The amount of TA was calculated by Folin–Ciocalteu’s method.^34^ About 100 μL of supernatant was mixed with 200 μL of Folin–Ciocalteu’s reagent (Merck, Germany) and vortexed for 30 s; then, 800 μL of Na_2_CO_3_ (Sigma-Aldrich, USA) 0.7 M in Milli-Q water was added and incubated for 2 h at room temperature (RT). The absorbance was read with a Varioskan Multimodal Plate Reader (Thermo Fisher Scientific, USA) at 765 nm.

The amount of Fe^3+^ was determined as follows.^35^ About 100 μL of supernatant was mixed with 100 μL of a solution of ammonium thiocyanate (Sigma-Aldrich, USA) 1 M and incubated for 15 min at RT. Then, the absorbance was measured with a Varioskan Multimodal Plate Reader at 490 nm. The amount of each compound was calculated by the difference between the total amount added for the coating and the amount quantified in the supernatant.

In vitro drug release was performed in a synovial-mimicking fluid,^33^ in sink conditions, at pH 5.5 and 7.4, to simulate a pro-inflammatory and physiological environment. 1% of Tween-80 (Sigma-Aldrich, USA) was used in the synovial-mimicking fluid to help the solubilization of curcumin for the quantification. Briefly, free drug as control, the drug-loaded, and the TA-coated NPs were immersed in the release medium (15 mL) at 37 °C and stirred at 150 rpm. About 200 μL of samples was taken after a specific time point, replaced by the same amount of fresh pre-warmed medium, and analyzed by HPLC, using the method described above. The cumulative percentage of drug release was calculated using eq 3
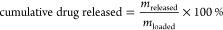
3where *m*_released_ is the mass of drug released (in mg) and *m*_loaded_ is the total amount of drug loaded (in mg).

### Cell Culture and Cytotoxicity Studies

Human primary tenocytes from the human extensor *indicis proprius* and KG-1 macrophages’ cell line (ATTC CCL-246) were used to assess the in vitro compatibility of the developed NPs. The isolation of human tenocytes is described in the Supporting Information.

DMEM (F-12, Gibco), supplemented with 10% of FBS (Gibco, USA), 1% of penicillin and streptomycin (PEST, Gibco, USA), and 200 μM of ascorbic acid (Sigma-Aldrich, USA), was used to grow tenocytes in an incubator (ESCO Celculture CO_2_ incubator, ESCO Scientific) at 37 °C, 5% of CO_2_, and 95% relative humidity.

IMDM (Sigma-Aldrich, USA), supplemented with 10% FBS, 1% PEST, and 1% non-essential aminoacidic (NEA, HyClone) solution, was used to grow KG-1 macrophages in an incubator (BB 16 gas incubator, Heraeus Instruments GmbH) at 37 °C, 5% of CO_2_, and 95% relative humidity.

The cytocompatibility of the nanosystems was assessed in both cell types. Briefly, cells were seeded in a 96-well plate (Corning, USA) at a density of 1 × 10^4^ cells per well, and primary tenocytes (passages #4 and #5) were left to attach overnight, while KG-1 cells (passage #13) were immediately incubated with particles. NPs in suspension were prepared in the respective medium at the final concentration of 50, 100, 250, and 500 μg/mL, and the complete medium and Triton X-100 (Merck Millipore, Darmstadt, Germany) were used as negative and positive controls. After 24 h of incubation (37 °C, 5% of CO_2_, and 95% relative humidity), 50 μL of CellTiter-Glo (Promega, USA) was added directly to KG-1; tenocytes were instead washed twice with Hank’s balanced salt solution-(N-[2-hydroxyethyl]piperazine-N′-[2-ethanesulfonic acid]) (HBSS–HEPES, pH 7.4), and then, 100 μL of HBSS–HEPES and CellTiter-Glo (1:1) was added to the cells. Finally, the luminescence was read with the Varioskan multimodal plate reader. The cytocompatibility of TA, Fe^3+^, and the TA-Fe^3+^ complex was also tested, and the concentrations used corresponded to the amount present on the surface of the TA-coated NPs (Figure S4).

### Quantitative Uptake Studies on Human Tenocytes

Human primary tenocytes (passages #4 and #5) were seeded into a 12-well plate (Corning, USA) at a density of 2 × 10^5^ cells per well and let to attach at 37 °C overnight. Then, cells were incubated with 50 μg/mL of particles (drug-loaded and TA-coated NPs), 1.4 μg/mL of curcumin, and DMEM-F12 as a negative control for 3 h.

After incubation, samples were washed twice with 500 μL of phosphate buffer saline (PBS)-ethylenediaminetetraacetic acid (EDTA), detached with trypsin (Cytiva, HyClone, USA), and washed twice again. Then, cells were collected in 500 μL of PBS in FACS tubes (Falcon, Corning Brand), and the uptake was evaluated by an LSRII flow cytometer (BD Bioscience, USA). To quench the external fluorescence of curcumin, 500 μL of trypan blue (TB; 0.005% v/v—Gibco USA) were added for 5 min, and then samples were resuspended in 300 μL of PBS–EDTA after centrifugation (5 min, 400*g*). The results were analyzed with FlowJo software v.10 (Tree Star, Inc., USA). The gating strategies of flow cytometry results are shown in Schemes S1–S3 in Supporting Information. Quantitative studies of uptake on KG-1 are described in Supporting Information.

### Mechanism of Uptake Studies in Human Tenocytes

The mechanism used by cells to uptake the NPs was studied in human primary tenocytes. Cells (passages #4 and #5) were seeded into a 12-well plate (Corning, USA) at a density of 2 × 10^5^ cells per well and let to attach at 37 °C overnight. Before adding NPs, cells were treated with selected pathway inhibitors (listed in Table S3) for 1 h and then incubated with 50 μg/mL of drug-loaded and TA-coated NPs for 3 h. After incubation, the cells were washed with PBS–EDTA, detached with trypsin, resuspended in 500 μL of PBS, and analyzed by LSRII as described above.

### Qualitative Uptake of Drug-Loaded and TA-Coated NPs in Human Tenocytes

Qualitative uptake of NPs was studied by confocal imaging in human tenocytes. Cells (passage #5) were seeded at a concentration of 3 × 10^5^ in an 8-well chamber (Thermo Fisher Scientific, USA) and allowed to attach overnight. 200 μL of samples (25 μg/mL of NPs and DMEM-F12 as a negative control) was added, and cells were incubated at 37 °C for 1 h. Then, cells were washed with PBS, and the cell membrane was stained with 200 μL CellMask Deep Red (50 ng/mL, Thermo Fisher, USA) for 3 min at 37 °C. Samples were washed with PBS, and cells were fixed with 200 μL of a solution of 4% (v/v) of PFA (Sigma-Aldrich, USA) for 15 min at 37 °C. After washing, the nucleus was stained with DAPI (Thermo Fisher Scientific, USA) at concentration of 2.5 μg/mL for 3 min, washed twice, and then stored in PBS at +4 °C. The images were captured with a Leica TCS SP8 STED 3X CW 3D Inverted Microscope (Leica Microsystems, Germany), using a 63× water objective, and analyzed with Leica AS software (Leica Microsystems, Germany).

### Anti-inflammatory and Anti-Fibrotic Studies on Human Tenocytes Using RT-qPCR

The anti-inflammatory effect of curcumin and the anti-fibrotic effect of TA were evaluated by RT-qPCR. Human primary tenocytes (passage #5) were seeded in a 12-well plate (Corning, USA) at a density of 1 × 10^5^ cells per well and allowed to attach overnight. Then, cells were treated with a solution of 250 ng/mL of LPS from *Escherichia coli* (O111:B4, InvivoGen, USA) to induce inflammation for 24 h. AcDEX, AcDEX TA-coated, drug-loaded, and TA-coated NPs, as well as curcumin, TA, and DMEM-F12 used as a control, were added to the cells for 24 h to achieve the maximum release of curcumin. Tenocytes treated with LPS were used as a positive control while cells with DMEM-F12 as a negative control. The RNA was isolated using TRIzol reagent (Ambion, USA) and Phase Lock Gel system (5PRIME, lock Gel heavy, QuantaBio), following the manufacturer’s instructions. The cDNA was synthesized using the First-strand cDNA Synthesis Kit (Transcriptor First strand cDNA synthesis kit, Roche, Germany), and finally, the RNA was analyzed with a LightCycler 480 qPCR machine (GE Healthcare Lifescience) with Taqman chemistry. The probes used in the assay were from Thermo Fisher Scientific and pre-designed: *18S* (4333760T), NF-κb (*Nfkb1*, Hs00765730_m1), TGF-β (*Tgfb1*, Hs00998133_m1), matrix metallopeptidase 3 (*Mmp3*, Hs00968305_m1), and matrix metallopeptidase 9 (*Mmp9*, Hs00957562_m1). The ΔΔCT of each sample was quantified, and the results were normalized to the housekeeping gene *18S*.

### Ethical Permissions

Patient’s recruitment, participation, and sample collection were obtained after receipt of a signed informed consent, approved by the Helsinki and Uusimaa Hospital District ethics committee (HUS/2785/2020) and by the institutional review board (HUS/234/2020).

### Statistical Analysis

The statistical analysis was performed in GraphPad Prism 9 (GraphPad Software, Inc., La Jolla, CA, USA). A detailed description of the statistical methods used to analyze the data is reported in each figure legend. In general, ordinary one-way ANOVA followed by a Dunnett post-hoc test, ordinary two-way ANOVA followed by a Dunnett post-hoc test, and a paired Student’s *t*-test were used for the statistical analyses of the different studies.

## Supporting Information Available

Additional synthesis of acetalated dextran, isolation of human tenocytes, TEM images for the morphological structure of the particles; optimization of the flow rates and speed of stirring used for particles preparation; quantitative uptake on KG-1 macrophages (M0 and M1 phenotypes), compounds used to inhibit the different mechanisms of endocytosis, different concentrations used to check the cell cytotoxicity of the uptake inhibitors in human tenocytes, LD % and efficiency of encapsulation (EE %) for drug-loaded and TA-coated NPs; chemical structures of dextran and AcDEX; drug-release profile of curcumin from drug-loaded and TA-coated NPs in a synovial-mimicking fluid at pH 5.5 and pH 7.4, up to 2 h; cytocompatibility of TA and iron on human tenocytes and KG-1 after 24 h of incubation; cytocompatibility of cell-uptake inhibitors after 4 h of incubation on human tenocytes; percentage of KG-1 cells presenting CD86 after 24 h; quantitative cell-uptake studies on KG-1 macrophages, M0 and M1 after 3 h of incubation with curcumin, drug-loaded and TA-coated NPs; gatings for the flow cytometry study of cells–NPs interaction in human tenocytes; gatings for the flow cytometry study of cells–NPs interaction in KG-1; and gatings for the flow cytometry study of the expressed marker CD86 in KG-1 after treatment with LPS and switching of phenotype from M0 to M1 (PDF)
