# Supplementary material for: In Vitro Study of the Anti-inflammatory and Antifibrotic Activity of Tannic Acid-Coated Curcumin-Loaded Nanoparticles in Human Tenocytes
Source: ACS Appl Mater Interfaces. 2023 May 2;15(19):23012–23. doi: 10.1021/acsami.3c05322 (PMC10197084; doi:10.1021/acsami.3c05322)
Supplement: Supplementary file 1 — am3c05322_si_001.pdf [file am3c05322_si_001.pdf]

SUPPORTING INFORMATION

An *In Vitro* Study of the Anti-inflammatory and Anti-fibrotic Activity of Tannic Acid-coated Curcumin-loaded Nanoparticles in Human Tenocytes

*Giuseppina Molinaro[1], Flavia Fontana[1], Rubén Pareja Tello[1], Shiqi Wang[1], Sandra López Cérda[1], Giulia Torrieri[1], Alexandra Correia[1], Eero Waris[2], Jouni T. Hirvonen [1], Goncalo Barreto [3,4,5], Hélder A. Santos[1,6,7,]**

[1] Drug Research Program, Division of Pharmaceutical Chemistry and Technology, Faculty of Pharmacy, University of Helsinki, P.O. Box 56, Fabianinkatu 33, FI-00014, Helsinki, Finland

[2] Department of Hand Surgery, University of Helsinki and Helsinki University Hospital, FIN-00029 HUS, Helsinki, Finland

[3] Translational Immunology Research Program, Faculty of Medicine, University of Helsinki, PL 4 (Yliopistonkatu 3), 00014, Helsinki, Finland

[4] Medical Ultrasonics Laboratory (MEDUSA), Department of Neuroscience and Biomedical Engineering, Aalto University, 02150, Espoo, Finland

[5] Orton Orthopedic Hospital, Tenholantie 10, 00280 Helsinki, Finland

[6] Department of Biomedical Engineering, University Medical Center Groningen, University of Groningen, Ant. Deusinglaan 1, 9713 AV Groningen, The Netherlands

[7] W.J. Kolff Institute for Biomedical Engineering and Materials Science, University Medical Center Groningen, University of Groningen, Ant. Deusinglaan 1, 9713 AV Groningen, The Netherlands

*Corresponding author: h.a.santos@umcg.nl

SUPPLEMENTARY MATERIALS AND METHODS

Synthesis of Acetalated Dextran (AcDEX).

AcDEX (**Figure S1**) was synthesized as described elsewhere.[1] Briefly, 1 g of dextran (MW: 9000-11000 kDa, Sigma-Aldrich USA) was dissolved in 10 mL of dimethylsulfoxide (DMSO, Sigma-Aldrich USA). Then 15.6 mg of pyridinium p-toluenesulfonate (Fluka, Assay≥99.0%) were added as a catalyst, followed by 3.4 mL of 2-methoxypropene(Sigma-Aldrich USA). After 2h the reaction was stopped with 1 mL of triethylamine (TEA, Sigma-Aldrich USA), and the product was precipitated with Milli-Q water, then centrifuged (35min, 154,324 *g*, Optima L-100 XP Ultracentrifuge, Beckman Coulter). The pellet was washed twice with Milli-Q water and dried by lyophilization (CoolSafe, Scanvac) overnight.

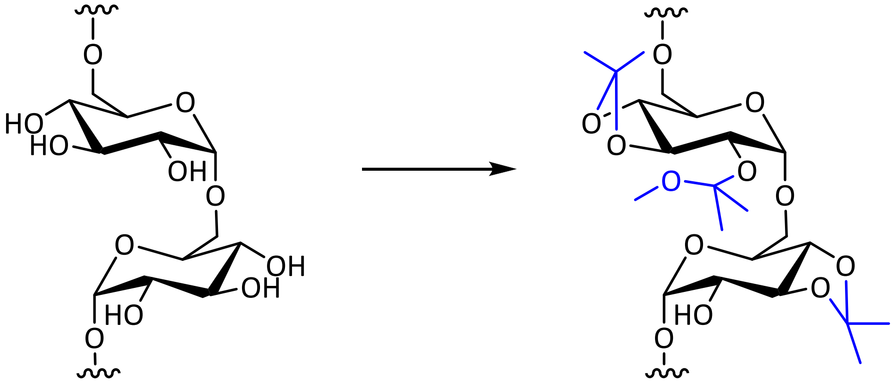

Figure S1. Chemical structures of dextran and AcDEX.

Isolation of Human Tenocytes from Human Extensor Indicis

The tendon sample was taken after surgery on a 58-year-old patient due to the rupture of the extensor *pollicis longus* tendon: the sample is derived from the extensor *indicis proprius*. The isolation of tenocytes was performed using the collagenase method. The tendon was collected in sterile PBS and then all the surrounding tissue sheath was removed using scissors and forceps. The tissue was washed once with PBS, then minced in small pieces (around 5 × 4 mm) and digested for 4 h in 20 mL of DMEM supplemented with 2 mg/mL of collagenase II at 37 °C.[2] Then cells were filtered with a 70 µm cell strainer and centrifuged at 400 × g for 10 min. Tenocytes were resuspended in the growth medium and attached to a cell culture flask.

Patient's recruitment, participation, and sample collection were obtained after receipt of a signed informed consent, approved by the Helsinki and Uusimaa Hospital District ethics committee (HUS/2785/2020) and by the institutional review board (HUS/234/2020).

Quantitative Uptake on KG-1 Macrophages (M0 and M1 phenotypes)

KG-1 macrophages (M0) were seeded into a 12-well plate (Corning, USA) at a density of 2×10^5 per well and then incubated with 50 μg/mL of particles (Drug-Loaded and TA-coated NPs), 1.4 μg/mL of curcumin and complete medium as a negative control for 3 h. After incubation, cells were centrifuged (438×*g*, 5 min), washed twice with PBS–EDTA, and collected in 500 μL of PBS in flow cytometry tubes (Falcon, Corning Brand). The uptake was evaluated by BD Accuri C6 Plus (BD, USA) flow cytometer. To quench the external fluorescence of curcumin, 500 μL of trypan blue (TB; 0.005% (v/v), Gibco USA) were added for 5 min and then samples were resuspended in 300 μL of PBS–EDTA after centrifugation (5min, 448×*g*). The results were analyzed with FlowJo™ software v.10 (Tree Star, Inc., USA). To activate KG-1 to the phenotype M1, 2×10^5 cells were seeded into a 12-wells plate and treated with a solution of lipopolysaccharide (LPS) 100 ng/mL for 24h. Then quantitative uptake of NPs was performed as described above. To confirm the switching of phenotype in macrophages, from M0 to M1, the expression of the costimulatory marker CD86 was measured after the treatment with LPS. The cells were centrifuged and stained with CD86-APC antibody (BD, Bioscience, USA - Lot.9179092), washed twice with PBS, and analyzed by BD Accuri C6 Plus (BD, USA) flow cytometer.

Supplementary Data

Table S1. Optimization of the flow rates and the speed of stirring used for the preparation of AcDEX NPs.

AcDEX	curcumin	flow rate inner phase	flow rate outer phase	speed of stirring	size	PDI
10 mg/mL	/	1 mL/hr	30 mL/hr	500 rpm	436.6 ± 3.4	0.47 ± 0.01
10 mg/mL	/	1 mL/hr	70 mL/hr	800 rpm	352.7 ± 13.5	0.12 ± 0.06
10 mg/mL	/	1 mL/hr	60 mL/hr	800 rpm	191.2 ± 21.9	0.19 ± 0.07
10 mg/mL	0.5 mg/mL	1 mL/hr	60 mL/hr	800 rpm	257.1 ± 33.7	0.18 ± 0.05

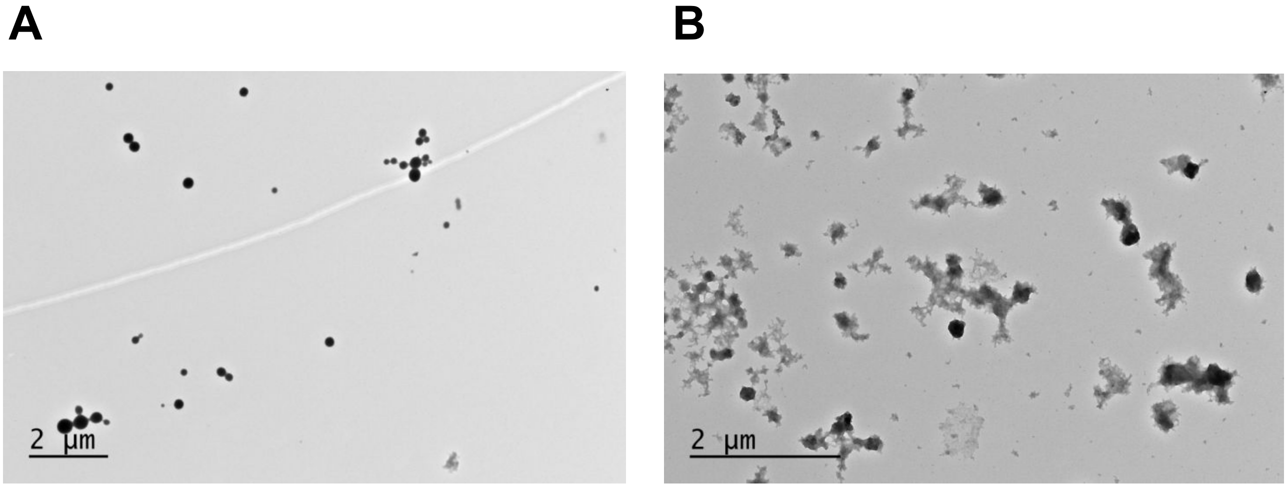

Figure S2. TEM images for the morphological structure of the particles before (A) and after (B) the TA-Coating. Scale bar = 2 µm.

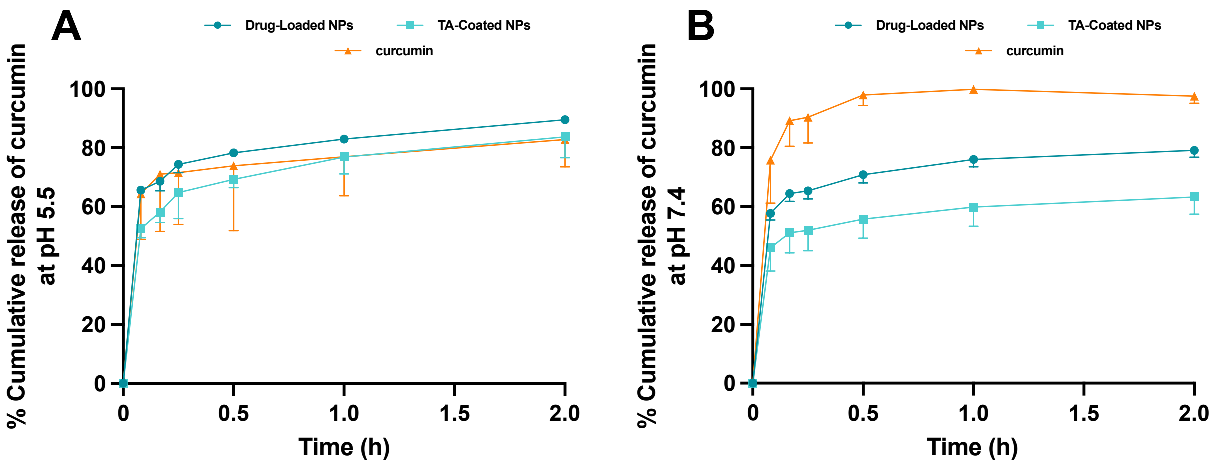

Figure S3. Evaluation of drug-release profile in sink conditions of curcumin from Drug-Loaded and TA-Coated NPs in synovial-mimicking fluid at (A) pH 5.5 and (B) pH 7.4, kept at 37°C and under stirring at 300 rpm up to 2 h. Results are presented as mean ± s.d. (n ≥ 3).

Table S2. Loading degree (LD%) and efficiency of encapsulation (EE%) for Drug-Loaded and TA-Coated NPs.

Formulation	LD %	EE %
Drug-Loaded NPs	4.3 ± 0.5	56.0 ± 4.4
TA-Coated NPs	2.8 ± 0.2	40.7 ± 5.5

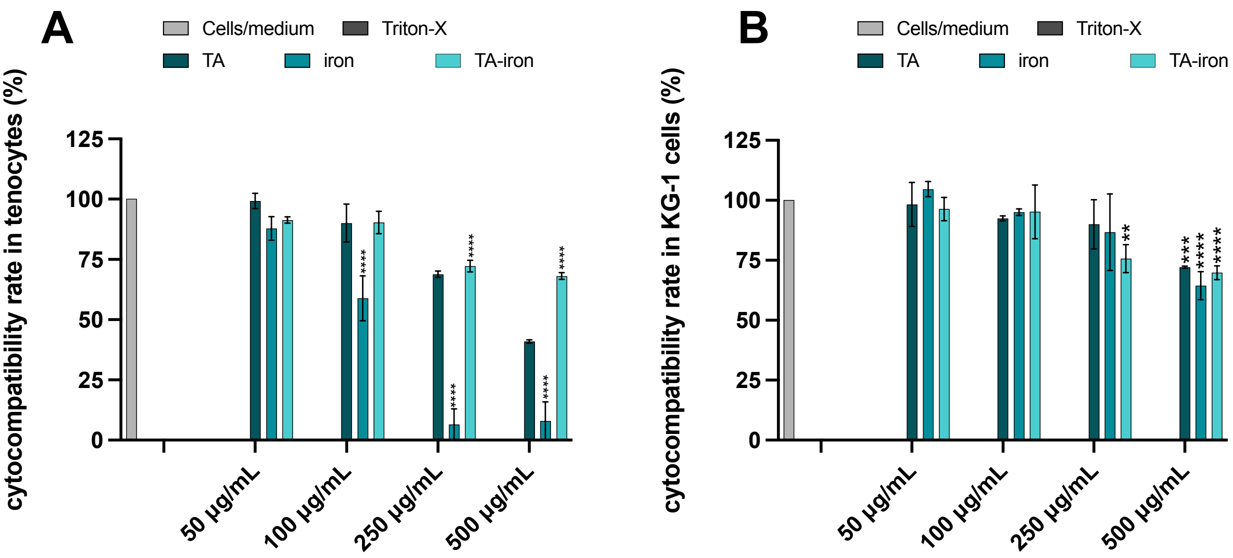

Figure S4. Cytocompatibility of TA and iron on (A) human tenocytes and on (B) KG-1 (M0) (after 24 h of incubation. Cell culture media and Triton-X 100 (1%) represented negative and positive control, respectively. The data are represented as the concentration of TA and iron present in 50 µg (TA: 12.4 µg and iron:1.6 µg), 100 µg (TA: 24.8 µg and iron:2.24 µg), 250 µg (TA: 62 µg and iron:5.6 µg) and 500 µg (TA: 124 µg and iron:11.2 µg) of TA-coated NPs. Results are presented as mean ± s.d. (n = 3) and the samples were analyzed with Ordinary one-way ANOVA, followed by a Dunnett post hoc test, setting the probabilities at ***p < 0.01,*** p < 0.001, and ****p < 0.0001.*

Studies of the mechanism of the quantitative uptake.

Table S3. Compounds used to inhibit the different mechanisms of endocytosis.

Compound	Mechanism Inhibited
**Nocodazole**<br>**(MedChem Express, MCE®)**<br>20 μM	Actin polarization/depolarization[3]
**Genistein**<br>**(Sigma-Aldrich, USA)**<br>400 μM	Caveolin-mediated endocytosis[4]
**Sucrose**<br>**(Sigma-Aldrich, USA)**<br>225 mM	Clathrin-mediated endocytosis[5]
**3-methyl-β-cyclodextrin**<br>**(Sigma-Aldrich, USA)**<br>2 mM	Cholesterol-dependent endocytosis[6]
**Sodium azide**<br>**(Sigma-Aldrich, USA)**<br>100 mM	Active transport[7]

Table S4. Different concentrations used to check the cell-cytotoxicity of the uptake inhibitors in human tenocytes.

Compound	Low concentration	Medium concentration	High concentration
**Nocodazole**	10 μM	20 μM	40 μM
**Genistein**	200 μM	400 μM	800 μm
**Sucrose**	225 mM	450 mM	900 mM
**3-methyl-β-cyclodextrin**	1 mM	2 mM	4 mM
**Sodium azide**	50 mM	100 mM	200 mM

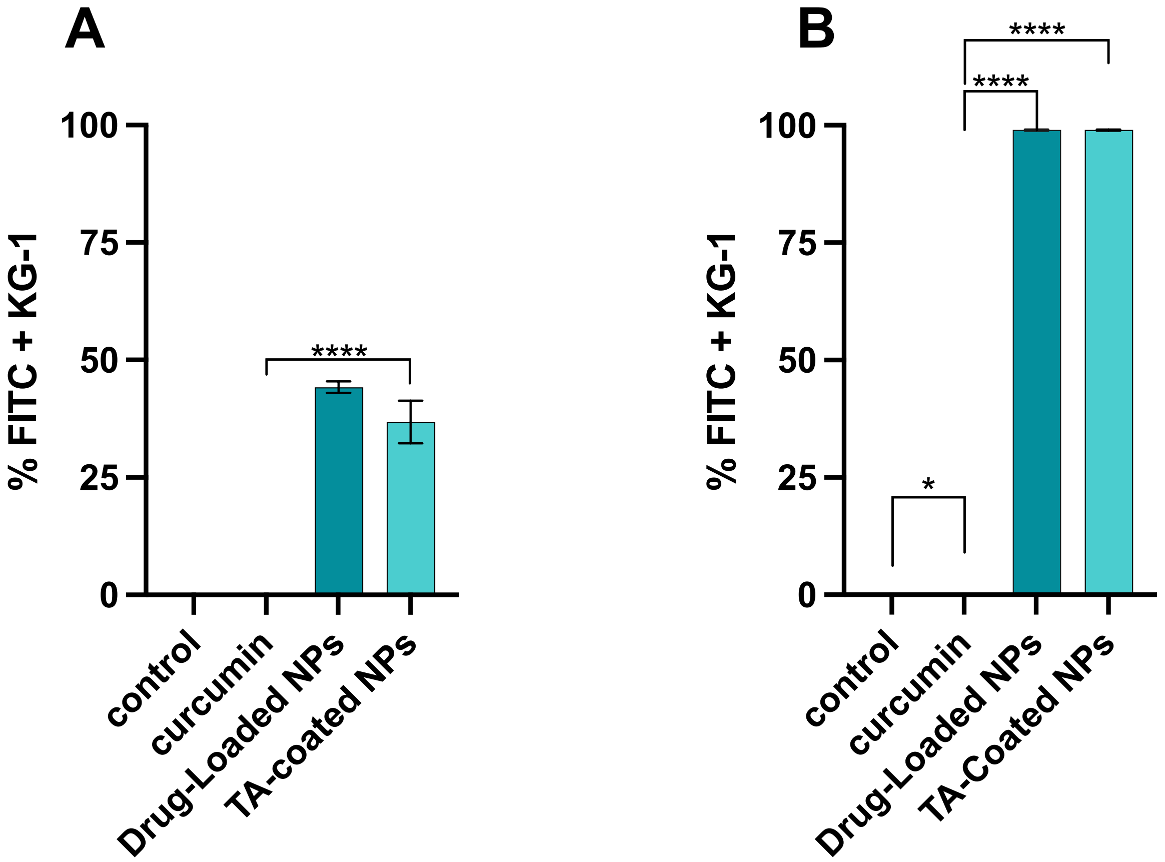

Figure S5. Quantitative cell uptake studies on (A) KG-1 macrophages (M0) and (B) KG-1 macrophages activated (M1). The cells were incubated for 3 h with curcumin, Drug-Loaded, and TA-Coated NPs and then the samples were analyzed by flow cytometer. Results are presented as mean ± s.d. (n = 3) and the samples were analyzed with Ordinary one-way ANOVA, followed by a Dunnett post hoc test, setting the probabilities $*p < 0.05$ and $****p < 0.0001$, comparing all the samples to the curcumin.

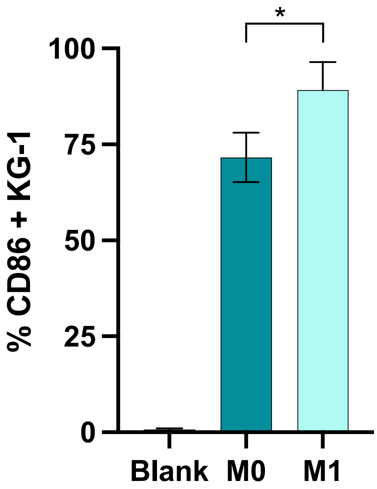

Figure S6. Percentage of KG-1 cells presenting CD86 after 24h. The cells were incubated with 100 ng/mL of LPS and then stained with APC-antihuman CD86 antibody. Results are presented as mean ± s.d. (n = 3) and the samples were analyzed with Ordinary one-way ANOVA, followed by a Dunnett post hoc test, setting the probabilities *$p < 0.05$*, comparing M0 to M1.

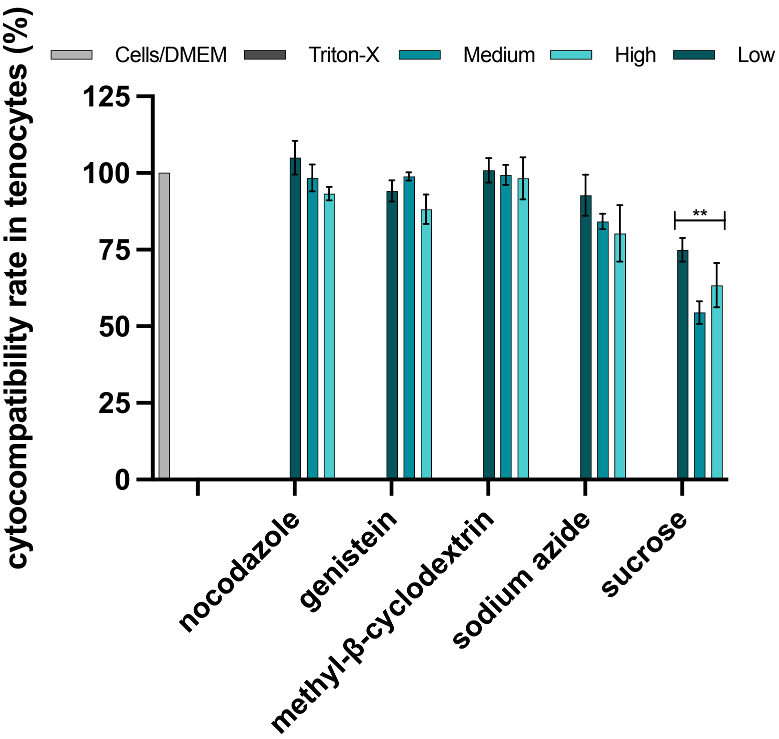

Figure S7. Cytocompatibility of cell-uptake inhibitors after 4 h of incubation on human tenocytes. Cell culture media and Triton-X 100 (1%) represented negative and positive control, respectively. Results are presented as mean ± s.d. (n = 3) and the samples were analyzed with Ordinary one-way ANOVA, followed by a Dunnett post hoc test, setting the probabilities at **$p < 0.01$.

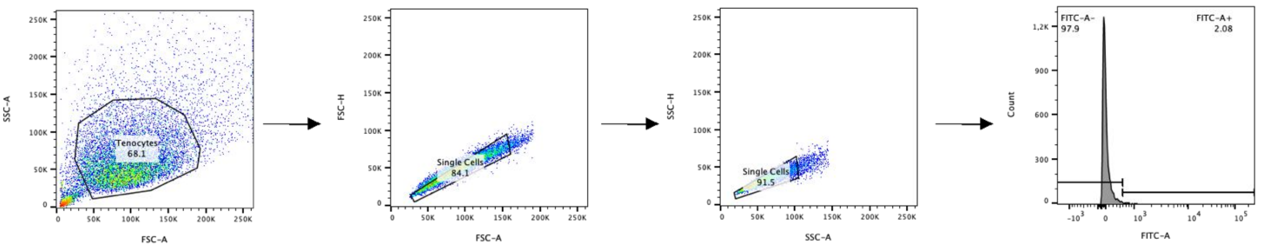

Scheme 1. Gatings for the flow cytometry study of cell-NPs interaction in human tenocytes.

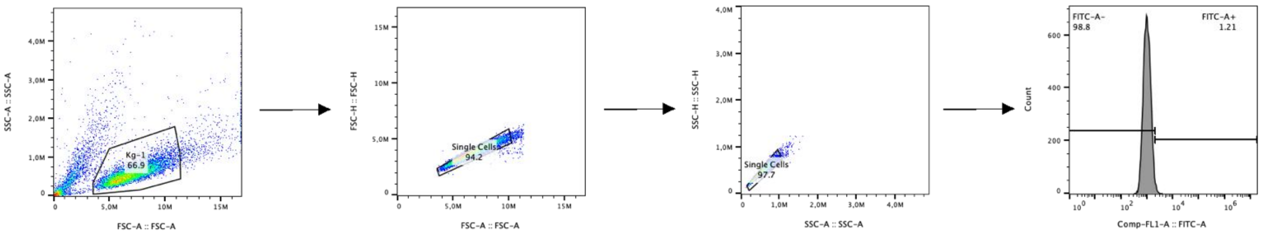

Scheme 2. Gatings for the flow cytometry study of cell-NPs interaction in KG-1.

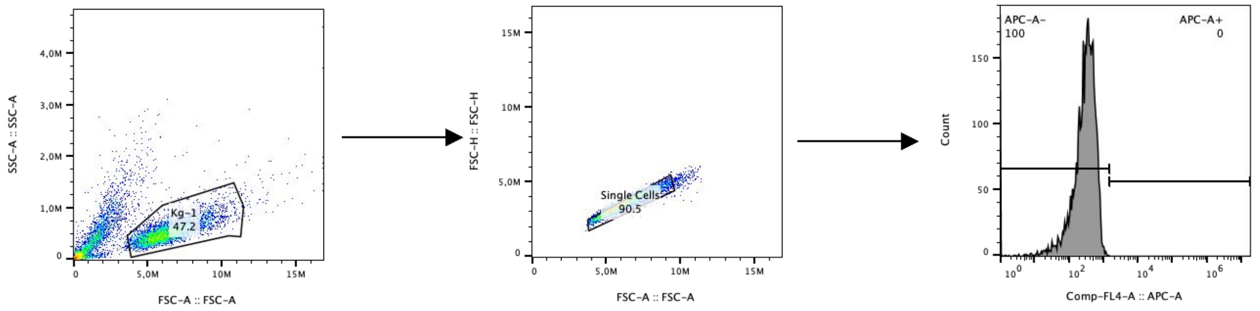

Scheme 3. Gatings for the flow cytometry study of the expressed marker CD86 in KG-1 after treatment with LPS and switching of phenotype from M1 to M2.

References

(1) Bachelder, E. M.; Beaudette, T. T.; Broaders, K. E.; Dashe, J.; Fréchet, J. M. Acetal-Derivatized Dextran: An Acid-Responsive Biodegradable Material for Therapeutic Applications. *J. Am. Chem. Soc.* **2008**, *130* (32), 10494-10495.
(2) Mazzocca, A. D.; Chowaniec, D.; McCarthy, M. B.; Beitzel, K.; Cote, M. P.; McKinnon, W.; Arciero, R. *In Vitro* Changes in Human Tenocyte Cultures Obtained From Proximal Biceps Tendon: Multiple Passages Result in Changes in Routine Cell Markers. *Knee Surg. Sports Traumatol. Arthrosc.* **2012**, *20* (9), 1666-1672.
(3) Zieve, G. W.; Turnbull, D.; Mullins, J. M.; McIntosh, J. R. Production of Large Numbers of Mitotic Mammalian Cells by Use of the Reversible Microtubule Inhibitor Nocodazole: Nocodazole Accumulated Mitotic Cells. *Exp. Cell Res.* **1980**, *126* (2), 397-405.
(4) Vercauteren, D.; Vandenbroucke, R. E.; Jones, A. T.; Rejman, J.; Demeester, J.; De Smedt, S. C.; Sanders, N. N.; Braeckmans, K. The Use of Inhibitors to Study Endocytic Pathways of Gene Carriers: Optimization and Pitfalls. *Mol. Ther.* **2010**, *18* (3), 561-569.
(5) Bhattacharyya, S.; Warfield, K. L.; Ruthel, G.; Bavari, S.; Aman, M. J.; Hope, T. J. Ebola Virus Uses Clathrin-Mediated Endocytosis as an Entry Pathway. *Virology* **2010**, *401* (1), 18-28.
(6) Yan, J.; Li, Q. F.; Wang, L. S.; Wang, H.; Xiao, F. J.; Yang, Y. F.; Wu, C. T. Methyl-β-Cyclodextrin Induces Programmed Cell Death in Chronic Myeloid Leukemia Cells and, Combined With Imatinib, Produces a Synergistic Downregulation of ERK/SPK1 Signaling. *Anticancer Drugs* **2012**, *23* (1), 22-31.
(7) Okano, T.; Yamada, N.; Okuhara, M.; Sakai, H.; Sakurai, Y. Mechanism of Cell Detachment From Temperature-Modulated, Hydrophilic-Hydrophobic Polymer Surfaces. *Biomaterials* **1995**, *16* (4), 297-303.
